# Randomized Clinical Trial Comparing Bucket Handle and Cartilage Tympanoplasty Techniques for the Reconstruction of Subtotal or Anterior Tympanic Membrane Perforation

**DOI:** 10.1155/2018/2431023

**Published:** 2018-05-22

**Authors:** Alimohamad Asghari, Mohammad Mohseni, Ahmad Daneshi, Yasser Nasoori, Sara Rostami, Maryam Balali

**Affiliations:** ^1^Skull Base Research Center, Iran University of Medical Sciences, Tehran, Iran; ^2^ENT and Head & Neck Research Center and Department, Hazrat Rasoul Akram Hospital, Iran University of Medical Sciences (IUMS), Tehran, Iran

## Abstract

**Objective:**

The purpose of the study is to compare the clinical outcome of the two techniques of Bucket Handle Tympanoplasty and Cartilage Tympanoplasty in achieving success in graft survival as well as acceptable auditory results. 60 patients who suffered chronic otitis media with anterior perforation of the tympanic membrane were chosen. The patients were randomly assigned using Block Randomization Method of two groups including patients who underwent Bucket Handle Tympanoplasty (*n* = 30) or those that underwent Cartilage Tympanoplasty (*n* = 30). The patients were followed up for 1, 3, 6, and 12 months postoperatively.

**Results:**

The mean PTA was lower in Bucket Handle Tympanoplasty group as case group compared to Cartilage Tympanoplasty group as the control (*P* = 0.023). No significant statistical differences had identified passing through the time, in terms of PTA outcome (*P* Value = 0.547) and SRT outcome (*P* Value = 0.352), between Bucket Handle Tympanoplasty group and the Cartilage Tympanoplasty group. In total, postoperative tympanic membrane perforation was found in 10.0% of patients in Cartilage Tympanoplasty group and 13.3% in Bucket Handle Tympanoplasty group with no difference (*P* = 0.500).

**Conclusions:**

Hearing improvements in both methods were similar.

**Registration Number:**

The trial is registered with IRCT2016022626773N1.

## 1. Introduction

Chronic otitis media is one of the most common and important middle ear disorders; it causes serious cost and resources implications for healthcare systems around the world particularly in developing countries. The prevalence of chronic otitis media in Southeast Asia, Africa, and Western Pacific countries is 2–4%, and that in North America and European countries is <2% [1]. Chronic otitis media is one of the main causes of tympanic membrane perforations which in effect leads to hearing loss and predisposing chronic infections. Tympanic perforations can heal over time; however, in order to achieve faster recovery and prevent further infective complication, surgical procedures may be advisable for repairing perforations and securing dry ear conditions [2, 3]. The purpose of the tympanoplasty surgical procedure is to create a dry and noninfected environment in the ear. Yet, many people suffering from chronic otitis media-related tympanic membrane perforations will have to undergo tympanoplasty procedures. Henceforth, it is crucial that the practitioner ensures due consideration is applied in order to minimize the complications to the procedure for the patient [4]. Tympanoplasty surgical procedure is conducted in two ways, namely, underlay and overlay [5]. It is important to note that the overlay techniques had been the preferred technique till the 1970s; however, due to complications such as lateralization of graft, delay in wound healing, and graft to repair, the general tendencies of surgeons and practitioners have gone towards underlay techniques [6]. Bucket Handle Tympanoplasty is one of the leading underlay techniques.

Since introducing tympanoplasty in 1952, various types of graft materials have been applied to reconstruct the impaired tympanic membrane such as cartilage, temporalis fascia, and fat or vein [7]. However, one of the problems with repairing the tympanic membrane is to maintain its anterior part after repair. This is because of the absence of proper vascular support in the anterior part of the canal which causes graft necrosis in that region, the absence of anatomical structures to hold the graft after placement in the proper place, and the angle between the tympanic wall and the anterior wall of the canal [8, 9]. In initial tympanoplasties, temporalis fascia was used with high success rate ranged from 93% to 97% [10]. Yet, within the last decade, the use of cartilage was taken into consideration as an alternative to temporalis fascia graft [11]. In recent years tissue engineering methods used for reconstruction of tympanic membrane perforations include scaffold materials, biomolecules, and cells. One of the biomolecules used for tympanic membrane reconstruction is bFGF (basic fibroblast growth factor). bFGF has some receptors in the epithelial layer and it facilitates the perforation closure. The application of different scaffold material soaked with bFGF achieved a higher and fasting healing rate and it also reduced the time of surgery and infectious of the middle ear [12].

In comparison to temporalis fascia the rigidity of cartilage has been considered as its positive feature leading good resistance to the retraction; however, it might adversely lead to a defective acoustic transferring system [13]. Nevertheless, employing Cartilage Tympanoplasty in repairing the anterior perforation of tympanic membrane led to the overall success rate 96.9% to 98.4% [14]. Overall, the choice of graft material is affected by different factors that include, amongst many, the size of the perforation, surgeon's experience, and the tympanic membrane status [15, 16]. Although both fascia and cartilage are commonly used, it is yet to be seen which material has superiority over the other. The present study aimed to compare the clinical outcome of the Bucket Handle Tympanoplasty technique (using temporalis fascia with underlay technique) and Cartilage Tympanoplasty in achieving success in graft survival as well as acceptable auditory results.

## 2. Materials and Methods

### 2.1. Ethical Approval

This study was approved by the Ethics Committee of the Iran University of Medical Science with the registration code:* IR.IUMS.REC.1394.1334968*.

### 2.2. Study Population

This randomized clinical trial is registered for the Iranian Registry of Clinical Trials (IRCT). The study was performed on patients that suffered chronic otitis media with anterior or subtotal (involving all 4 quadrants and reaching up to annulus fibrosus) perforations of the tympanic membrane. The tympanoplasty procedures were carried out by a single surgeon in Tehran, Iran, at the Rasoul Akram Hospital in 2016.

The patients were randomly assigned to two groups using Block Randomization Method, in order to reduce bias and achieve balance in the allocation of participants to biases arms. The two groups included patients that underwent Bucket Handle Tympanoplasty (as case group) or those who underwent Cartilage Tympanoplasty (as the control group).

Block Randomization Method assigns equal number of participants within blocks of each treatment. Participants were both randomly identified and selected by ordering as well as assigning the specified groups and the participants underwent the operation on the basis of the sequences of the device. A key benefit of blocked randomization is that the treatment groups will be equal to the size and will tend to be uniformly distributed by key outcome related characteristics.

All patients were informed of their type of surgery and signed informed consent to their respective surgery. They were able to request to be excluded from the study if they were reluctant to take part in the study. The exclusion criteria were those with previous ear operations, evidence of cholesteatoma or retractable pocket without perforation, middle ear polyp, detachment or tightening of the ossicular chain, nasal allergy, cleft palate, intracranial complications, or need for mastoidectomy. In addition, the statistician who analyzed the results was blinded to the procedures, but all patients and surgeons were informed about the type of surgery.

Audiometry was performed according to the American Speech-Language-Hearing Association guidelines, 2005, with an audiometer Amplaid. Pure tone thresholds (PTA) were measured at frequencies of 250 to 8000 Hz. PTA was obtained using an ascending-descending method in 5 dB steps. Threshold was defined as the lowest decibel hearing level at which responses occur in at least one-half of a series of ascending trials. The minimum number of responses needed to determine the threshold of hearing is two responses from three presentations at a single level.

Speech reception threshold (SRT) was measured using an ascending-descending method in 5 dB steps. Six numbers of spondaic words (which are 2-syllable words that have equal stress on both syllables) were presented in each presentation level. Words were presented by live voice through microphones. In speech detection score, a total of 25 monosyllabic words were presented at most comfortable level.

### 2.3. Surgical Technique

**Figure 1:**
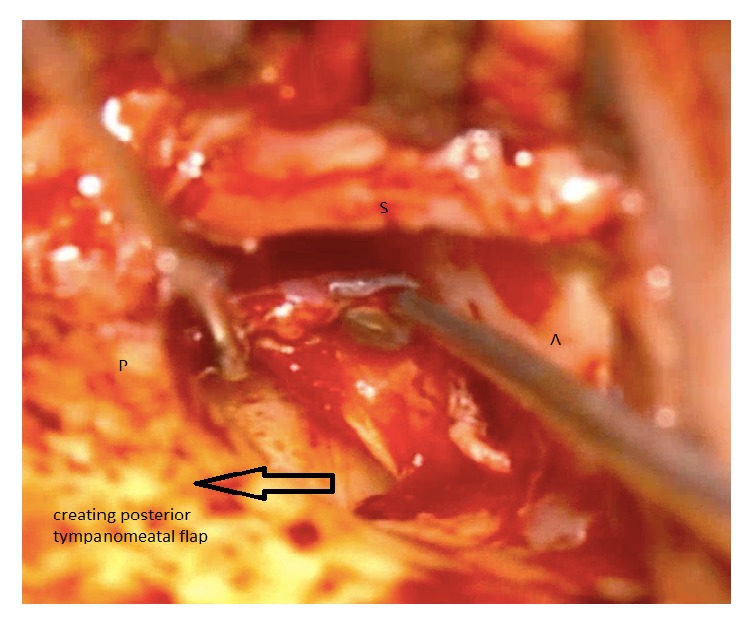
Elevation of the posterior tympanomeatal flap: A = anterior, P = posterior, and S = superior.

**Figure 2:**
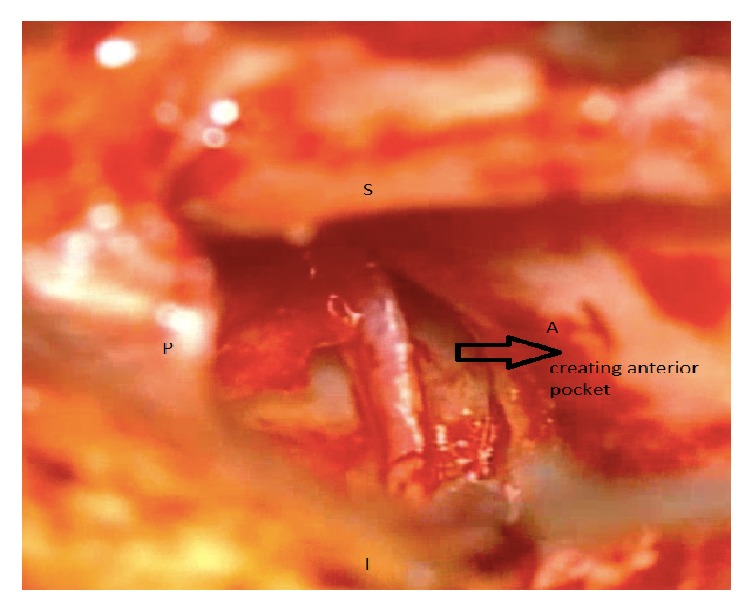
Elevation of the anterior tympanomeatal flap.

**Figure 3:**
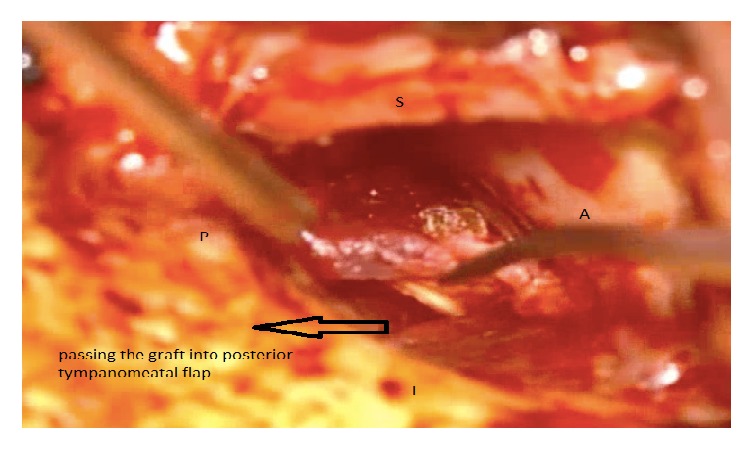
Passing the graft into posterior tympanomeatal flap.

**Figure 4:**
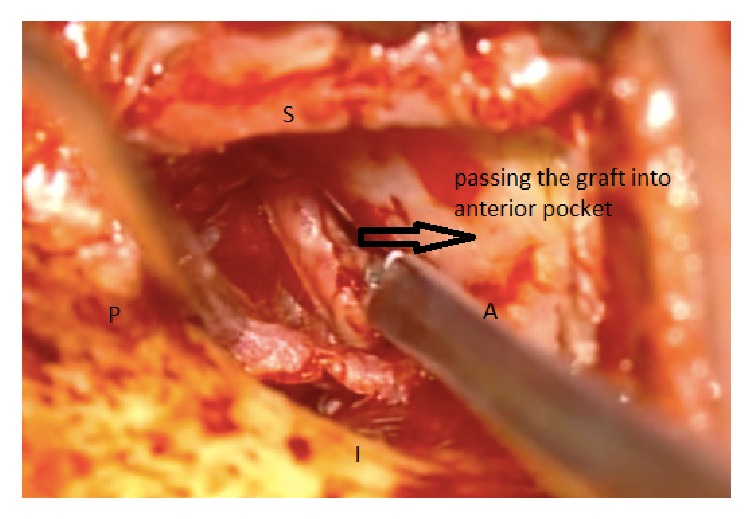
Passing the graft into the anterior pocket.

**Figure 5:**
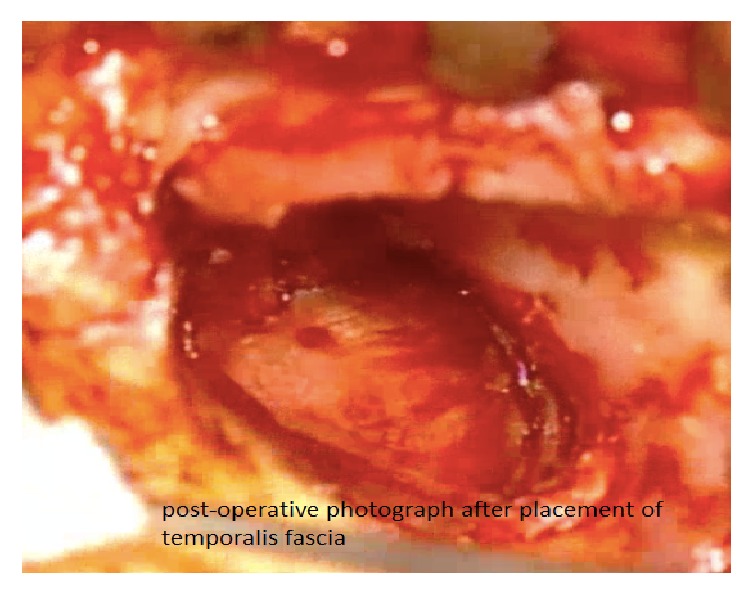
Final result after placement of the graft.

The two techniques included Bucket Handle Tympanoplasty and Cartilage Tympanoplasty. In Bucket Handle Tympanoplasty technique, first, the posterior tympanomeatal flap was raised. Then the anterior flap of the skin canal was created by cutting 3-4 mm lateral to the annulus and extended up to 1 mm higher and lower than the perforation edge and the annulus was removed from its groove. The graft from the deep fascia temporalis was implanted as underlying after freshening up the edges of the perforation. The middle ear was filled with gelfoam and then the graft was fixed under the anterior annular flap and the posterior tympanomeatal flap, and it is placed medial to the handle of malleus. After that the external auditory canal filled with gelfoam. Figures 1–5 and the Supplemental Digital Content show the steps of Bucket Handle Tympanoplasty procedure as described in surgical techniques.

In Cartilage Tympanoplasty technique, the cartilage was sliced into a thickness of 0.4 mm with a slicer and was fixed under the freshened edges of perforation and medial to the handle of malleus after filling the middle ear with gelfoam. After that, the external auditory canal filled with gelfoam.

Both surgeries are done with a microscope.

### 2.4. Patients' Follow-Up

All subjects were followed at 1, 3, 6, and 12 months after surgeries regarding audiometry to assess air-bone gap, SRT, SDS (speech discrimination score), and ear examination with a microscope to assess the persistence of graft and possible complications by regular visiting.

### 2.5. Statistical Analysis

Results were presented as the mean ± standard deviation (SD). The Chi-square test was used to assess the statistical significance of graft uptake and the repeated measures ANOVA test was performed to compare the hearing outcomes (SDS, SRT, and PTA) of the two groups. For the statistical analysis, the statistical software SPSS version 16.0 was used. *P* values of 0.05 or less were considered statistically significant.

## 3. Results

A total of 60 patients took part in the study. There were 30 patients in Cartilage Tympanoplasty control group and 30 patients in Bucket Handle Tympanoplasty case group. In the control group 15 cases were female (50%) and 15 (50%) were male. In the case group, 18 cases were female (60%) and 12 (40%) were male. The mean age in the control was 43.6 ± 3.3 and this figure was 44.7 ± 4.1 in the case group.

In the control group, 10 (33.3%) operations were done on the right ear and 20 (66.7%) on the left ear, whereas in the case group 13 (43.3%) operation were done on the right side and 17 (56.7%) on the left side. In control group 25 (83.3%) cases had anterior perforation and 5 (16.7%) had a subtotal perforation of tympanic membrane; in the case group 25 (83.3%) cases had anterior perforation and 5 (16.7%) had a subtotal perforation of the tympanic membrane. It was noted that the two groups were similar in gender distribution (*P* = 0.436), average age (*P* = 0.884), ear under operation (0.426), and the site of membrane perforation (*P* = 1.000). General characteristics of these patients are shown in Table 1. The repeated measures ANOVA test was used to assess the mean of PTA (at four frequencies of 500 Hz, 1000 Hz, 2000 Hz, and 4000 Hz) and SRT and SDS.  Table 2 shows the audiometry results at different time intervals.

*In Association with PTA.* There have been significant differences, in each of the Buckets Handle Tympanoplasty (*P* Value < 0.001) and Cartilage Tympanoplasty (*P* Value < 0.001) groups during the time.

Bucket Handle Tympanoplasty group had a significant statistical difference between before surgery and 3 months after surgery (*P* Value < 0.001), 6 months after surgery (*P* Value < 0.001), and 12 months after surgery (*P* Value < 0.001). But no differences were seen between 3 months after surgery and 6 months after surgery (*P* Value = 1), 3 months after surgery and 12 months after surgery (*P* Value = 1), and 6 months after surgery and 12 months after surgery (*P* Value = 1).

Cartilage Tympanoplasty group had significant statistical differences between before surgery and 3 months after surgery (*P* Value < 0.001), 6 months after surgery (*P* Value < 0.001), and 12 months after surgery (*P* Value < 0.001). But no differences were seen between 3 months after surgery and 6 months after surgery (*P* Value = 1), 3 months after surgery and 12 months after surgery (*P* Value = 0.417), and 6 months after surgery and 12 months after surgery (*P* Value = 1).

**Figure 6:**
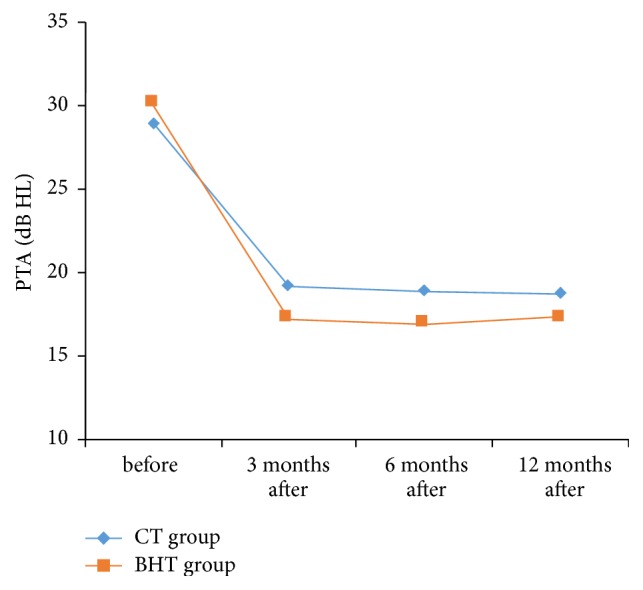
The trend of the change in PTA parameter in two groups.

According to Figure 6, no significant statistical differences had identified passing through the time, in terms of PTA outcome, between Bucket Handle Tympanoplasty group and the Cartilage tympanoplasty group (*P* Value = 0.547).

*In Association with SRT.* There had seen significant differences in each of the Bucket Handle Tympanoplasty (*P* Value < 0.001) and Cartilage Tympanoplasty (*P* Value < 0.001) groups during the time.

Bucket Handle Tympanoplasty group had significant statistical differences before surgery with 3 months and after surgery (*P* Value < 0.001), 6 months after surgery (*P* Value < 0.001), and 12 months after surgery (*P* Value < 0.001). But no differences were seen between 3 months after surgery and 6 months after surgery (*P* Value = 0.965), 3 months after surgery and 12 months after surgery (*P* Value = 0.965), and 6 months after surgery and 12 months after surgery.

Cartilage Tympanoplasty group had significant statistical differences between before surgery and 3 months after surgery (*P* Value < 0.001), 6 months after surgery (*P* Value < 0.001), and 12 months after surgery (*P* Value < 0.001). But no differences were seen between 3 months after surgery and 6 months after surgery (*P* Value = 1), 3 months after surgery and 12 months after surgery (*P* Value = 1), and 6 months after surgery and 12 months after surgery.

**Figure 7:**
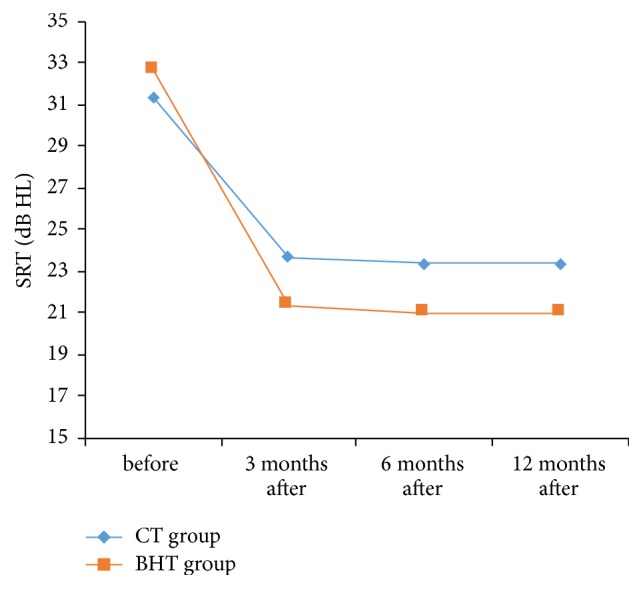
The trend of the change in SRT parameter in two groups.

According to Figure 7, no significant statistical differences had identified passing through the time, in terms of SRT outcome, between Bucket Handle Tympanoplasty group and the Cartilage tympanoplasty group (*P* Value = 0.352).

*In Association with SDS.* Bucket Handle Tympanoplasty group had no significant statistical differences between before surgery and 3 months after surgery (*P* Value = 0.109), 6 months after surgery (*P* Value = 0.109), and 12 months after surgery (*P* Value = 0.109).

Also, Cartilage Tympanoplasty group had no significant statistical differences between before surgery and 3 months after surgery (*P* Value = 0.965), 6 months after surgery (*P* Value = 0.965), and 12 months after surgery (*P* Value = 0.965).

**Figure 8:**
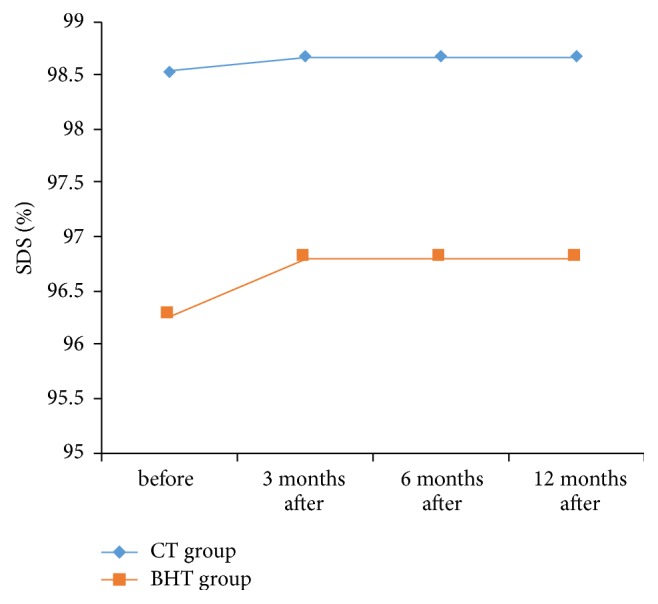
The trend of the change in SDS parameter in two groups.

According to Figure 8, significant statistical differences had identified passing through the time, in terms of SDS outcome, between Bucket Handle Tympanoplasty group and Cartilage tympanoplasty group (*P* Value = 0.022).

In total, postoperative tympanic membrane perforation was found in 10.0% of patients in the control group and 13.3% in case group with no significant difference (*P* = 0.500).

## 4. Discussion

Considering that the anterior and subtotal perforations of the tympanic membrane following the methods of repair have a higher degree of treatment failure, by reducing the therapeutic failure of these perforations, more favorable outcomes of the treatment of chronic otitis media are predictable. In a study by Hosamani et al. [17] on anterior and subtotal perforations, the success rate of anterior tagging of temporalis fascia material was considerably higher in comparison with other underlay methods (95% versus 5%).

In another survey by Hay and Blanshard [18] on 150 anterior and subtotal perforations, they used anterior pocket to support the anterior portion of the graft and the success rate of the procedure was revealed to be 91%, while in a similar trial by Kumar et al. [19], the success rate of Bucket Handle Tympanoplasty for repairing tympanic membrane perforation was shown to be 80%. Goycoolea et al. [20] in their book published in 1989 described a technique to overcome the problem of the anterior tympanic membrane perforation. In their technique, by creating a cut on the skin of the outer ear canal, a small anterior flap with a lateral base was inserted leading to a success rate of 94% for repairing membrane defect.

In Hashemi et al. [21] studies, the mean SRT and air-bone gap at four frequencies between 500 Hz, 1000 Hz, 2000 Hz, and 4000 Hz significantly improved in those who underwent Cartilage Tympanoplasty or tympanoplasty with temporalis fascia. Also, the difference in the air-bones gap before and after the operation was 17, 15, 15, and 18 after Cartilage Tympanoplasty and 19, 18, 19, and 23 after tympanoplasty with temporalis fascia. They also showed that the average of SRT improvement after one-year follow-up time was 17.9 ± 2.0 and 20.6 ± 2.0 in tympanoplasty with temporalis fascia and cartilage techniques, respectively.

One of the successful methods in repairing the anterior perforation of the tympanic membrane is Cartilage Tympanoplasty method with the success rate ranging from 98.4% to 96.9%. In a study by Ozbek et al. [22], the success rate achieved by Cartilage Tympanoplasty was 91% with significant decrease in air-bone gap (less than 20 dB) in 94%. Furthermore, Glasscock and Hart [23] employed repairing a perforation of the tympanic membrane using Cartilage Tympanoplasty method of the overall failure rate of 4%. In the systematic review and meta-analysis by Jeffery et al., they found that Palisade Cartilage Tympanoplasty has an overall take rate of 96% at beyond 6 months and has similar odds of complications compared to temporalis fascia. The air-bone gap closure is statistically similar to the reported results from temporalis fascia tympanoplasty [24].

The aforementioned studies all have utilized only one technique of tympanoplasty, whereas in our study we have utilized and compared the results of two tympanoplasty techniques, namely, Bucket Handle (using temporalis fascia) and Cartilage Tympanoplasty. Our study showed that the success rates in the closure of the tympanic perforation with use of temporalis fascia with the Bucket Handle technique are similar to use of cartilage. This can be because of the proper technique used in Bucket Handle Tympanoplasty, in which anterior support is provided. PTA and SRT in both groups had significant differences in improvement of hearing result after surgery. But 3 months after surgery no more improvement had been seen in groups; that means that the maximum result could be reached 3 months after surgery. Probably this can be due to the absorption of gelfoms of the middle ear and external auditory canal and eliminating inflammation of surgery. Cause of similarity of PTA and SRT in both groups could be due to slicing the cartilage to 4 mm thick, which makes it more structurally similar to fascia and tympanic membrane. Regarding the SDS, as we expected, changes in each group over time were not meaningful, and that is because the SDS most indicate the condition of the auditory nerve. But in our study, SDS changes over time between groups had been signed for the cartilage group; this can be because SDS in the cartilage group was higher since the beginning. It could also be due to small sample size.

### 4.1. Limitation of Study

This study has several limitations, our study was limited to adults, and 30 patients in each group were studied. Thus, future investigation into large studies would need to accurately compare this two methods. In our study, some conditions like cholesteatoma, retraction pocket, and Eustachian tube dysfunction have been excluded from the study. Also, we have to note that our study did not include total tympanic membrane perforation cases. All mentioned items have a different effects on the result of surgeries and further studies are required in future. Studies with long-term follow-up are also recommended to check graft survival and long-term auditory results.

Tissue engineering methods progress for reconstruction of tympanic membrane perforations. Fibroblast growth factor is one of the most investigated biomolecules that achieved improvement in tympanic closure. The use of this growth factor with suitable scaffold materials or even with fascia or cartilage graft may proceed to improvement in tympanic closure. And this can be seen in future studies.

## 5. Conclusion

Since the results of auditory tests and graft survival results in both groups were not statistically and clinically different, and according to the studies mentioned in the Cartilage Tympanoplasty shown that this technique is very reliable for restoration of tympanic membrane, therefore, Bucket Handle technique can be used as a good therapeutic modality for reconstruction of anterior and subtotal tympanic membrane perforations.

## Figures and Tables

**Table 1:** Baseline characteristics of study population.

Item	Cartilage Tympanoplasty	Bucket Handle Tympanoplasty	*P* value
Gender			0.436
Male	15 (50.0%)	12 (40.0%)	
Female	15 (50.0%)	18 (60.0%)	
Mean age	43.6 ± 3.3	44.7 ± 4.1	0.884
Ear under operation			0.426
Right	10 (33.3%)	13 (43.3%)	
Left	20 (66.7%)	17 (56.7%)	
Site of membrane perforation			1.000
Anterior	25 (83.3%)	25 (83.3%)	
Subtotal	5 (16.7%)	5 (16.7%)	

**Table 2:** The change in PTA, SRT, and SDS parameter in both groups within the follow-up time.

Item	Cartilage Tympanoplasty	Bucket Handle Tympanoplasty
PTA	Mean ± standard deviation	Mean ± standard deviation
Before surgery	28.92 ± 7.47 dB HL	30.08 ± 11.69 dB HL
3 months after surgery	19.17 ± 4.66 dB HL	17.17 ± 7.98 dB HL
6 months after surgery	18.83 ± 3.87 dB HL	16.92 ± 6.48 dB HL
12 months after surgery	18.67 ± 3.90 dB HL	17.25 ± 6.76 dB HL
SRT	Mean ± standard deviation	Mean ± standard deviation
Before surgery	31.33 ± 7.30 dB HL	32.67 ± 9.80 dB HL
3 months after surgery	23.67 ± 5.07 dB HL	21.33 ± 6.81 dB HL
6 months after surgery	23.33 ± 3.56 dB HL	21.00 ± 5.93 dB HL
12 months after surgery	23.33 ± 3.56 dB HL	21.00 ± 5.93 dB HL
SDS	Mean ± standard deviation	Mean ± standard deviation
Before surgery	98.53 ± 2.40%	96.27 ± 2.70%
3 months after surgery	98.67 ± 2.43%	96.80 ± 3.99%
6 months after surgery	98.67 ± 2.43%	96.80 ± 3.99%
12 months after surgery	98.67 ± 2.43%	96.80 ± 3.99%

## Data Availability

The data used to support the findings of this study are available from the corresponding author upon request.
